# Epidemiology of HIV among US Air Force Military Personnel, 1996–2011

**DOI:** 10.1371/journal.pone.0126700

**Published:** 2015-05-11

**Authors:** Shilpa Hakre, Dariusz G. Mydlarz, Peter Dawson, Patrick J. Danaher, Philip L. Gould, Catherine T. Witkop, Nelson L. Michael, Sheila A. Peel, Paul T. Scott, Jason F. Okulicz

**Affiliations:** 1 United States Military HIV Research Program, Henry M. Jackson Foundation for the Advancement of Military Medicine, Bethesda, Maryland, United States of America; 2 Preventive and Occupational Medicine Branch, National Guard Bureau, Arlington, Virginia, United States of America; 3 Biostatistics, The EMMES Corporation, Rockville, Maryland, United States of America; 4 Infectious Disease Service, San Antonio Military Medical Center, Fort Sam Houston, San Antonio, Texas, United States of America; 5 Air Force Medical Support Agency, Defense Health Headquarters, Falls Church, Virginia, United States of America; 6 United States Military HIV Research Program, Walter Reed Army Institute of Research, Bethesda, Maryland, United States of America; 7 HIV Diagnostics and Reference Laboratory, United States Military HIV Research Program, Walter Reed Army Institute of Research, Silver Spring, Maryland, United States of America; University of Washington, UNITED STATES

## Abstract

**Objective:**

The objectives of this study were to describe the epidemiology of HIV in the United States Air Force (USAF) from 1996 through 2011 and to assess whether socio-demographic characteristics and service-related mobility, including military deployments, were associated with HIV infection.

**Methods:**

We conducted a retrospective cohort analysis of USAF personnel who were HIV-infected during the study period January 1, 1996 through December 31, 2011 and a matched case-control study. Cases were USAF personnel newly-diagnosed with HIV during the study period. Five randomly-selected HIV-uninfected controls were matched to each case by age, length of service, sex, race, service, component, and HIV test collection date. Socio-demographic and service-related mobility factors and HIV diagnosis were assessed using conditional logistic regression.

**Results:**

During the study period, the USAF had 541 newly diagnosed HIV-infected cases. HIV incidence rate (per 100,000 person-years) among 473 active duty members was highest in 2007 (16.78), among black/ African-American USAF members (26.60) and those aged 25 to 29 years (10.84). In unadjusted analysis restricted to personnel on active duty, 10 characteristics were identified and considered for final multivariate analysis. Of these single (adjusted odds ratio [aOR], 8.15, 95% confidence interval [CI] 5.71–11.6) or other marital status (aOR 4.60, 95% CI 2.72–7.75), communications/ intelligence (aOR 2.57, 95% CI 1.84–3.60) or healthcare (aOR 2.07, 95% CI 1.28–3.35) occupations, and having no deployment in the past 2 years before diagnosis (aOR 2.02, 95% CI 1.47–2.78) conferred higher odds of HIV infection in adjusted analysis.

**Conclusion:**

The highest risk of HIV infection in the USAF was among young unmarried deployment-naïve males, especially those in higher risk occupation groups. In an era when worldwide military operations have increased, these analyses identified potential areas where targeted HIV prevention efforts may be beneficial in reducing HIV incidence in the USAF military population.

## Introduction

Although the overall HIV incidence in the United States Air Force (USAF) is reported annually and comparable to that of the United States (US) military,[1] a comprehensive description of human immunodeficiency virus type 1 (HIV-1) in the USAF has not been described since early in the epidemic.[2] Simulation tools to inform decision-making in HIV and sexually transmitted disease (STD) prevention and control have used individual-level epidemiological, demographical, behavioral, and other factors to develop models reflective of the transmission dynamics of HIV and other STDs in populations.[3,4] These modeling studies have shown mobility as “an important risk factor in HIV transmission and control”.[5,6] Mobility in these and other studies considered the effects of migration (immigration/emigration) and short-term work- or relaxation-related travel on HIV/STDs.[7] Conversely, other reports indicate mobility has been associated with decreased risk of HIV or increased access to services.[8,9]

Historically, US military personnel have been at increased risk of STDs during deployments including World War II and other conflicts.[10] More recently, in surveys from the 1990’s, deployed US Navy and Marine Corps personnel and US military personnel stationed overseas who reported sexual contact with commercial sex workers had increased risk of STDs.[11,12] Among US military personnel deployed to Iraq or Afghanistan from 2004 to 2009, an examination of electronic laboratory records revealed incidence rates of *Chlamydia* increased during the study period.[13] Furthermore, in the same conflict, an investigation among US Army soldiers diagnosed with HIV on post-deployment screening indicated one soldier may have acquired HIV infection while deployed.[14]

The impact of mobility, including deployments and changes in duty station, on the incidence of HIV infection in the USAF has not been described in the past decade. From September 2001 through December 2011, the USAF has deployed at least 309,000 troop-years among active duty personnel in support of military operations conducted in Iraq and Afghanistan.[15] The objectives of this study were to describe the epidemiology of HIV in the USAF from 1996 through 2011 and to assess whether socio-demographic characteristics and service-related mobility were associated with HIV infection. Findings from these analyses will contribute to prevention and intervention programs in the USAF with the goal of reducing the incidence of HIV and STDs in our population. To assist in the development of HIV prevention and care programs and policies, the Centers for Disease Control and Prevention(CDC) recommends development of an epidemiologic profile of a population from socio-demographic, geographic, and other characteristics.[16] We conducted a retrospective cohort analysis of HIV-infected USAF members and a matched case-control study to accomplish these study objectives.

## Methods

Since early 1986, the USAF, in keeping with Department of Defense (DoD) policy, has implemented compulsory HIV testing among applicants to USAF service and periodic and peri-deployment testing among its active and reserve personnel. All active duty personnel diagnosed with HIV infection, including activated USAF National Guard and Reservists, are medically evaluated centrally at the USAF HIV Medical Evaluation Unit located at Joint Base San Antonio, Texas.[17]

The study population consisted of all USAF personnel on active service or in the Reserve or National Guard at any time during the study period, January 1, 1996 through December 31, 2011 who had demographic data available. Among those eligible, anyone newly-diagnosed with HIV during the study period was considered a case. Five randomly-selected controls were matched to each case by age, length of service (±3 months), sex, race, service, component, and HIV test collection date or index date (in a 30-day interval of a case’s first HIV-positive blood sample collection date). The 1:5 case-to-control ratio was chosen to ensure 80% or greater power to estimate a population odds ratio of 1.5 in risk factor analyses.[18]

### Ethical Considerations

The Walter Reed Army Institute of Research’s Division of Human Subjects Protection Office (#1898) made a determination that the study was minimal risk human subjects’ research. All personal identifying information for the study population was stripped and assigned individual study identification numbers before the datasets were sent to investigators for analyses.

### HIV Diagnosis

HIV diagnosis was determined by Food and Drug Administration (FDA)-approved HIV assays. In general, initial screening was conducted with an enzyme-linked immunoassay (ELISA) or an enzyme immunoassay (EIA). Assays used through the study period included Vironostika HIV-1 Microelisa System (bioMerieux, Durham, North Carolina, US), HIV AB HIV-1/HIV-2 (rDNA) EIA (Abbott Laboratories, Abbott Park, Illinois, US) and, currently, the ADVIA Centaur HIV 1/O/2 Enhanced (EHIV) (Siemens Healthcare Diagnostics, Tarrytown, New York, US). Samples that were screen-reactive were repeated in duplicate with the same assay. If two of three test results were reactive, the sample was reflexed to FDA-approved supplemental/confirmatory assays, HIV-1 Western Blot (Genetic Systems HIV-1 Western Blot, Bio-Rad Laboratories, Redmond, Washington, US) and/or an immunofluorescent antibody (IFA) test (Fluorognost HIV-1 IFA, Waldheim Pharmazeutika, GmbH, Vienna, Austria).

### Data Collection

Longitudinal data for the study population were obtained from the USAF School of Aerospace Medicine (USAFSAM) HIV Testing Service (USAFTS) at Wright-Patterson Air Force Base and the Armed Forces Health Surveillance Center (AFHSC), which maintain HIV testing history database and the Defense Medical Surveillance System (DMSS), respectively.[19] The USAFTS provided last negative and initial positive HIV test dates identifying newly HIV-diagnosed USAF personnel during the study period. The AFHSC provided longitudinal records for HIV testing, duty assignment, deployment, and socio-demographic characteristics for the entire study population as well as annual force strength (person-time) data for the Air Force.

### Data Analysis

Incidence rates were used to describe the epidemiology of HIV in the Air Force from 1996 through 2011; rates were calculated using a generalized linear model with a log link and the person-time denominator considered all subjects at risk in the Air Force (i.e. all USAF personnel who were HIV-uninfected during the study period). The relationship between socio-demographic and service-related factors and HIV infection was assessed in univariate (unadjusted) analysis using conditional logistic regression to account for matching between cases and controls. Cases missing a factor were excluded from analysis as were all matched controls. Socio-demographic factors were extracted at the index date and included marital status, highest level of education attained, occupation status, rank, home of record, and country of birth. Service-related factors chosen for study consisted of changes in duty assignments, specifically at the regional and zip code levels, number of deployments prior to, and during the two years before, the index date, and the total duration of deployments prior to the index date. A final multivariate model of factors potentially associated with HIV infection was determined using forward and backward iterative stepwise selection; a criteria p-value of 0.10 and 0.01 were used to enter and retain factors in the model, respectively. All analyses were performed using SAS version 9.3 (Cary, North Carolina).

## Results

**Fig 1:**
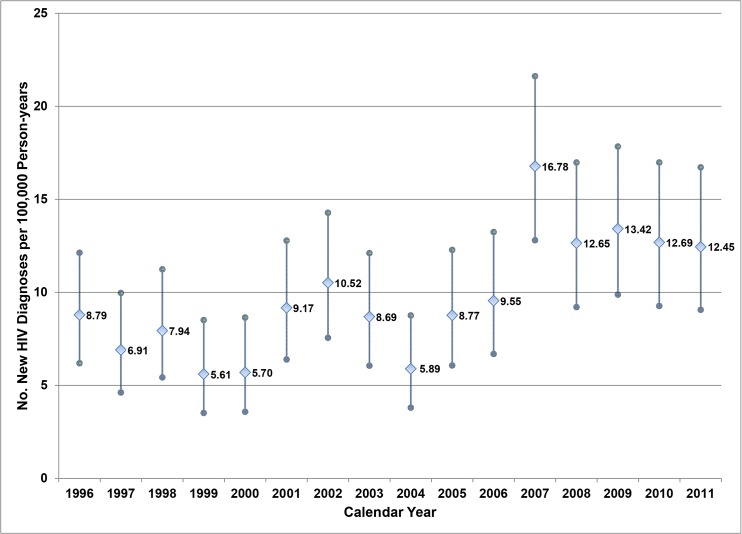
Incidence rates of new HIV diagnoses (and 95% confidence intervals) among active duty Air Force personnel, 1996–2011.

Among eligible USAF personnel, the USAFTS identified 541 newly diagnosed HIV-infected cases among 569,703 Air Force personnel who served between January 1, 1996 and December 31, 2011. Cases were predominantly active duty males aged 29 years or younger (Table 1). The 541 cases were almost equally of black/African American (n = 245, 45%) and white (n = 255, 47%) racial origin in number, however, overall incidence rate was highest among blacks/African Americans (21.14 per 100,000 person-years) and five-fold higher when compared to whites (incidence rate ratio [IRR] 5.31,95% confidence interval [CI], 4.45–6.32). Among active duty members, HIV incidence rate was highest in 2007 (Fig 1) and reached statistical significance when compared to the HIV incidence rate in 1996 (IRR, 1.91, 95%CI, 1.25–2.92, p = 0.003).

**Table 1:** Frequencies and incidence rates of HIV among United States Air Force personnel in service at any time from 1996 through 2011*.

Characteristic	No. HIV Positive	Incidence Rate per 100,000 Person Years	Incidence Rate Ratio (IRR)	95% CI Around IRR	P-value
Component					
Active Duty	473	8.40	Referent	-	-
National Guard	30	1.75	0.21	(0.14–0.30)	<0.001
Reserve	38	3.31	0.39	(0.28–0.55)	<0.001
**Active Component Only**					
Sex					
Male	463	10.14	10.79	(5.77–20.18)	<0.001
Female	10	0.94	Referent	-	-
Race**					
White	215	5.15	Referent	-	-
Black	223	26.6	5.17	(4.28–6.23)	<0.001
Other	35	13.38	2.6	(1.82–3.72)	<0.001
Age (years)					
20 or younger	28	4.81	Referent	-	-
21–24	126	9.93	2.06	(1.37–3.11)	<0.001
25–29	133	10.84	2.25	(1.50–3.39)	<0.001
30–34	77	8.21	1.71	(1.11–2.63)	0.015
35–39	66	7.34	1.53	(0.98–2.37)	0.061
45 or older	10	4.83	1.00	(0.49–2.07)	0.993

*All personnel with available demographic records were included in this analysis.

**Other race included Asians or Pacific Islanders, American Indians or Alaskan natives, and individuals who self-reported their race as ‘Other’ on their personnel records.

For risk factor analysis, 2,600 controls were matched to 541 cases by age, length of service, sex, race, component, and HIV test date. All controls met the pre-determined ranges for matching except for 3 individuals whose HIV negative date was within a 70-day interval of the respective case’s HIV positive date instead of a 30-day interval. Since only 68 of 541 cases (13%) were members of the Reserve and National Guard components and overall unadjusted HIV incidence rates were 2.6 to 4.8 times lower compared to active duty personnel, the final risk factor analysis was restricted to active duty personnel who included 473 cases (Table 1) and their matched controls. The mean length of military service for cases with available data (n = 305) was 8.26 years (range 0.00–36.57 years, interquartile range 3.09–12.54 years) at the time of HIV diagnosis. In unadjusted analyses, demographic characteristics of being unmarried (single or other marital status), having some college level education or less, having an enlisted rank, and having an occupation in communications, intelligence, or healthcare, conferred a significantly higher odds of HIV infection (p<0.05, Table 2). Also in unadjusted analyses for mobility-related characteristics, USAF personnel who had never deployed or those who had not deployed within the past 2 years, personnel having 1 or fewer changes in duty assignments ever or those who had no duty assignment changes in the past 2 years had greater odds of HIV infection (p<0.05, Table 3).

**Table 2:** Socio-demographic factors of HIV-infected and matched HIV-uninfected United States Air Force active duty personnel, 1996–2011.

Characteristic	Cases(n = 473)	Controls(n = 2315)	Unadjusted Odds Ratio	95% Confidence Interval	P-Value
Marital status*					
Married	94	1297	Referent	-	-
Other	46	142	4.21	(2.78–6.38)	<0.0001
Single, never married	333	873	9.76	(7.20–13.2)	<0.0001
Missing	0	3	-	-	-
Highest education attained					0.0074
Bachelors or higher	59	401	Referent	-	-
Some college or less	412	1890	1.71	(1.23–2.38)	0.0013
Missing	2	24	-	-	-
Occupation* *					
Communications/ Intelligence	170	494	2.84	(2.14–3.76)	<0.0001
Engineer/ Mechanic/ Repair	94	757	Referent	-	-
Healthcare	48	170	2.20	(1.47–3.29)	0.0001
Other	146	742	1.57	(1.16–2.14)	0.0036
Pilot/ aircrew	15	152	0.73	(0.41–1.31)	0.2875
Rank					
Enlisted	430	1982	1.95	(1.36,2.80)	0.0003
Officer	43	333	Referent	-	-
Home of Record^					
Continental US	304	1405	Referent	-	-
Other/ unknown	76	318	0.87	(0.59,1.30)	0.5055
Missing	93	592	-	-	-

*Other marital status included individuals who reported that they were neither married nor single on their personnel records.

**The occupation category, engineer, included non-combat engineer; ‘Other’ occupations included infantry/ combat engineer/ Special Forces/ artillery/ armor/ motor transport/ administration and other categories.^A service member’s residence at the time of entrance to the US military.

**Table 3:** Mobility-related factors among HIV-infected and matched HIV-uninfected United States Air Force active duty personnel, 1996–2011.

Characteristic	Cases(n = 473)	Controls(n = 2315)	Unadjusted Odds Ratio	95% Confidence Interval	P-Value
Region of assignment					<0.0001
America—South	268	953	1.70	(1.32–2.18)	<0.0001
Americas—West	104	621	Referent	-	-
Europe	29	218	0.80	(0.52–1.25)	0.3323
Non-US—Americas	6	31	1.05	(0.43–2.60)	0.9112
Pacific	18	131	0.85	(0.50–1.44)	0.5420
US—other	42	300	0.81	(0.55–1.19)	0.2772
Missing	6	61	-	-	-
Region changes					0.0049
1	113	674	0.89	(0.67–1.17)	0.3982
2 or more	205	1032	Referent	-	-
None	155	609	1.37	(1.04–1.82)	0.0270
Assignment /Permanent Station Changes (Zip Code)					0.0956
0–1	136	591	1.25	(0.96–1.62)	0.0956
2 or more	337	1724	Referent	-	-
Number of prior deployments					<0.0001
Deployed	171	1041	Referent	-	-
None	302	1274	1.62	(1.28–2.05)	<0.0001
Total deployments during prior 2 years					<0.0001
1 or more	90	690	Referent	-	-
None	383	1625	1.95	(1.50–2.54)	<0.0001

In multivariate analysis, active duty USAF personnel in communications/ intelligence (odds ratio [OR], 2.57, 95% CI, 1.84–3.60), healthcare (OR, 2.07, 95% CI, 1.28–3.35) occupations, of single (OR, 8.15, 95% CI, 5.71–11.6) or other marital status (OR, 4.60, 95% CI, 2.72–7.75), and having no deployment in the past 2 years before diagnosis (OR, 2.02, 95% CI, 1.47–2.78) had higher odds of HIV infection after adjusting for the factors above (Table 4).

**Table 4:** Characteristics retained in multivariate conditional logistic regression model.

Factor	Comparison	Adjusted Odds Ratio	95% CI	P-value
Total deployments during prior 2 years	None vs. one	2.02	(1.47–2.78)	<0.0001
Marital status	Other vs. married	4.60	(2.72–7.75)	<0.0001
	Single vs. married	8.15	(5.71–11.6)	<0.0001
Occupation	Communication/ intelligence vs. engineer/ mechanic/ repair	2.57	(1.84–3.60)	<0.0001
	Healthcare vs. engineer/ mechanic/ repair	2.07	(1.28–3.35)	0.0030
	Other vs. engineer/ mechanic/ repair	1.60	(1.12–2.28)	0.0097
	Pilot/ aircrew vs. engineer/ mechanic/ repair	0.83	(0.30–2.27)	0.7148

## Discussion

Our analyses of more than a decade of data suggest target areas for HIV prevention efforts. Active duty Air Force personnel who were young or of black/African American racial origin had the highest overall HIV incidence rate from 1996 through 2011. HIV incidence rate among active duty USAF personnel peaked in 2007 and dropped to a stable but higher rate than observed in the previous decade. USAF personnel who were not married or had certain occupations or had no deployments in the two years prior to diagnosis had the highest odds of HIV infection in matched case-control analysis after adjustment for multiple factors.

An increase in incidence rate of HIV diagnosis seen in the study period may be due to changes in HIV testing policy from previous years. On March 29, 2004 the Armed Forces Epidemiology Board recommended standardizing HIV testing intervals across all Services.[20] Parallel to revised HIV testing recommendations by the CDC in 2006 [21] which proposed routine opt-out screening in healthcare settings for persons aged 13 to 64, the DoD issued an instruction for HIV in October 2006 to all Services to screen active duty and activated military personnel every two years [22] resulting in the USAF implementing a force-wide increase in uptake of HIV testing. Furthermore, in 2007, the Air Force Testing Laboratory, the central HIV testing laboratory for the USAF, revised the existing HIV testing algorithm to replace a second-generation HIV assay with a more sensitive third-generation assay (Advia Centaur XP analyzer (HIV1/O/2 Enhanced (EHIV)). Conversely, despite an increased uptake in HIV testing in the US, HIV incidence rates (per 100,000 population) decreased by 33.2% from 24.1 in 2002 to 16.1 in 2011.[23]

A previous study describing HIV trends from 1986 to 1990 in the USAF reported markedly higher overall HIV incidence rates among non-white active duty men aged 20 to 29.[2] A similar trend persisted in our unadjusted analysis; active duty men aged 21 to 29 had the highest incidence as did active duty men of black or other race whereas active duty women had an overall near zero incidence. Furthermore, after adjusting for other factors, unmarried (single or other marital status) USAF active duty personnel had the highest odds of infection compared to those who were married. This risk profile is similar to the national trends where incidence has increased among younger men who have sex with men (MSM). Despite an overall decrease in HIV incidence rates in the US from 2002 to 2011, among males aged 13 to 24, HIV incidence rates per 100,000 population increased from 12.5 in 2002 to 17.3 in 2011; new HIV diagnoses increased in the US among men 24 years or younger, especially those in the MSM transmission category.[23] While a 2010 RAND report estimated that 30% of military service members participated in high-risk sexual activity that met CDC’s criteria for annual HIV testing,[24] self-disclosed sexual risk behavior among USAF personnel remains to be reported post repeal of the Don’t Ask Don’t Tell (DADT) policy in 2011.[25] However, 81% of HIV-infected males in the USAF followed for mandatory routine clinical evaluations reported same sex or bisexual behavior which suggests MSM sexual transmission as the primary driver of the HIV epidemic in the USAF.[26]

Factors related to occupation such as operational tempo, to include non-deployment periods, hazards unique to the military, and trauma, have been associated with higher sexual and other risk behaviors in a study among Belizean military personnel. [27] Dia et al. reported higher rates of occupational exposure to blood and body fluids among non-hospital healthcare providers among French military personnel.[28] In the US Navy, HIV incidence rates varied among Sailors in different occupations (or ratings).[29] In our analyses, USAF active duty personnel in communications, intelligence, or healthcare independently had higher odds of HIV infection. In general, people with better education and better cognitive ability have healthier behaviors and therefore better health outcomes.[30] Technical and intelligence occupations in the USAF require an entrance Armed Forces Vocational Aptitude Battery (ASVAB) of 95 or higher. However, despite expected healthier outcomes based on education and cognition, USAF personnel in communications or intelligence had greater odds of HIV infection which suggests other factors likely contribute to HIV risk in USAF personnel serving in these career fields. These factors deserve further study.

Previous studies in the US Army and US Navy reported peri-deployment to be a period of higher risk-taking as evidenced by detection of acute HIV infections in retrospective testing of HIV sero-negative samples collected from deployment-experienced HIV-infected personnel.[14,29] However, in an unadjusted analysis in a joint US Army-USAF case control study, which was limited to HIV cases identified from 2000–2004, fewer changes in duty locations and having deployments were found to be protective for HIV infection although the two factors were not independently associated with HIV infection. HIV genotyping of cases identified in that four-year period indicated 97% of HIV infections acquired by US Army and USAF personnel were HIV-1 subtype B,[31] the predominant subtype circulating in the US.[32] In our current analyses which spanned 16 years, having no deployments in the prior two years was associated with higher odds of HIV infection after adjustment for multiple factors. While a description of HIV subtyping is in progress for all new USAF cases, the null finding of mobility conferring higher odds of HIV infection combined with the predominance of subtype B among USAF personnel infected from 2000–2004 suggest infections are acquired primarily in the US.

While HIV incidence among active duty USAF is stable, our analyses delineate specific subgroups for targeted prevention efforts. Although our analyses did not include behavioral and clinical factors that may contribute to the understanding of HIV epidemiology among USAF personnel, young unmarried deployment-naïve males, especially those in higher risk occupation groups, may benefit from more frequent HIV testing than the biennial compulsory force testing currently in place. Early diagnosis and treatment result in improved health outcomes in HIV-infected individuals, such as improved immune reconstitution and better prognosis. Okulicz et al. reported that in a cohort of HIV-infected US military health system beneficiaries, individuals who were treated within 12 months of their estimated sero-conversion date had better CD4 recovery, lower risk for acquired immunodeficiency syndrome (AIDS), and better response to hepatitis B vaccine, versus those treated after 12 months.[33] Providing a mechanism for higher risk individuals to self-identify (e.g. on an annual health assessment) would allow for targeted education and more frequent testing as indicated. In a recent US Air Force study, Matthews et al. reported the interval between the last negative and first positive HIV tests was a median of 17.5 months (range, 10.7 to 26.1).[17] Since service personnel have universal access to care, reported barriers to receiving health services in the HIV care continuum are minimal.[34] In the continuum of diagnosis, care, and treatment, the US Air Force has been very successful in offering linkage to care and treatment services to HIV-infected personnel; a majority of HIV-infected personnel had an initial medical evaluation for HIV within a month of diagnosis (median 39 days) and a majority (88.1%) achieved viral load suppression (<50 copies/ml) within 12 months and 87.6% maintained suppression at 24 months.[17] Increased testing frequency and transition to a fourth generation test for earlier identification of infection could be added to an already successful HIV program to address possible unmet health needs of US Air Force personnel at highest risk of HIV. The CDC recommends, *at minimum*, annual testing for HIV and STDs in high risk groups such as sexually active MSM, MSM or heterosexuals with multiple partners, sex partners of HIV-infected individuals, and other groups.
